# Dietary Diversity and Food Variety in Chinese Children Aged 3–17 Years: Are They Negatively Associated with Dietary Micronutrient Inadequacy?

**DOI:** 10.3390/nu10111674

**Published:** 2018-11-05

**Authors:** Liping Meng, Yan Wang, Ting Li, Carolien Annika van Loo-Bouwman, Yumei Zhang, Ignatius Man-Yau Szeto

**Affiliations:** 1 Inner Mongolia Dairy Technology Research Institute Co., Ltd., Inner Mongolia, 63 Xidawang Road, Chaoyang District, Beijing 100022, China; mengliping@yili.com (L.M.); wangyan0@yili.com (Y.W.); liting2012@yili.com (T.L.); 2 Yili Innovation Center, Inner Mongolia Yili Industrial Group Co., Ltd., 8 Jinshan Road, Hohhot, Inner Mongolia 010110, China; 3 Yili Innovation Center Europe, Bronland 12E-1, 6708 WH Wageningen, The Netherlands; carolien.vanloo@yili-innovation.com; 4 School of Public Health, Peking University Health Science Center, 38 Xueyuan Road, Haidian District, Beijing 100191, China; zhangyumei@hsc.pku.edu.cn

**Keywords:** dietary diversity, food variety, micronutrient adequacy, children

## Abstract

Micronutrient inadequacy remains a nutritional problem in Chinese children. However, the associations between dietary diversity and inadequate micronutrient intake have not been extensively studied. A total of 2012 children aged 3–17 years from the China Health and Nutrition Survey were included for analysis. Dietary diversity score (DDS) and food variety scores (FVS) were assessed based on three 24-h recall periods. The nutrient adequacy ratio (NAR) was used to determine the micronutrient adequacy of the diet. The mean adequacy ratio (MAR, %) was defined as the sum of each NAR divided by the number of involved micronutrients. Overall micronutrient inadequacy (OMI) was defined as having a MAR below 0.75. Micronutrient inadequacy was defined as the proportion of individuals whose nutrient intake was less than the estimated average requirement. After adjustment confounders, DDS and FVSs were positively associated with MAR and NAR of most nutrients except sodium (*p* < 0.05). A higher DDS was negatively associated with the prevalence of inadequate intake of vitamin A, riboflavin, vitamin C, iron, zinc, selenium, niacin, phosphorus, magnesium and OMI. Similar results were found for FVSs. In conclusion, this study indicates that poor dietary diversity and food variety in Chinese children are directly associated with inadequate micronutrient intake.

## 1. Introduction

The nutritional status of Chinese children has improved over the past decades because of rapid social and economic development [1,2]. However, undernutrition and micronutrient inadequacy remain important nutritional problems for Chinese children. According to a national survey in 2010–2013, the national prevalence of stunting was 8.1% for children aged 0–5 years and 3.2% for those aged 6–17 years, while it was even higher in rural areas (11.3% and 4.7%, respectively). The prevalence of anemia was 5.0% and 8.0% for children aged 6–11 years and 12–17 years in China, respectively [3]. The general prevalence of serum vitamin D deficiency was 53.2% in Chinese children aged 6–17 years [4]. Unbalanced dietary patterns and inadequate dietary intakes appear to be common in Chinese children, with national data showing that the range of inadequate dietary intake rates of thiamin, riboflavin, vitamin C, and selenium varied from 53.9% to 91.5%, while dietary calcium inadequacy rates were as high as 97% among Chinese children aged 4–17 years [5]. Furthermore, Chinese children are facing a huge gap to meet the recommendation for dietary diversity and food variety. The latest Chinese Dietary Guidelines recommend that pre-school and school-aged children should consume at least 12 different food items for food variety and eight food groups for dietary diversity per day, which included cereals and potatoes, vegetables, fruits, legumes and nuts, meat and poultry, fish, eggs and milk [6]. Zhao et al. reported that Chinese children only consumed four of eight recommended food groups on average [7]. Since most micronutrients are obtained from the daily diet, a balanced diet containing diverse foods is essential. 

Several studies in Asia have reported that dietary diversity can be a valuable indicator to predict macronutrient or micronutrient adequacy for children [7,8,9]. The latest study in India (among adolescents) found that the nutrient adequacy ratio (NAR) of most micronutrients studied increased with the dietary diversity score (DDS), allowing them to conclude that nutrient adequacy was positively associated with high dietary diversity [8]. In a study conducted in the Philippines, DDS was found to be both significantly correlated to the mean probability of adequate nutrient intake (MPA) and to the intake of most micronutrients. For example, the Pearson’s correlation coefficient for MPA, vitamin A, riboflavin and folate was 0.36, 0.37, 0.33 and 0.30, respectively, indicating that dietary diversity was a significant predictor of adequate micronutrient intake of young non-breast-feeding Filipino children [9]. Lower dietary diversity in adults was also reported to be associated with increased risk of Type 2 diabetes [10,11] and cognitive impairment [12]. To date, the association between dietary diversity and food variety, with adequate micronutrient intake in Chinese children, has not yet been adequately studied.

Obtaining detailed quantitative data of dietary intake can be time consuming and expensive, and requires a high level of technical skill both in data collection and analysis, limiting its broad application. By contrast, dietary diversity is a qualitative measure of food consumption and has been recommended by the Food and Agriculture Organization (FAO) as a proxy for nutrient adequacy of the diet of individuals. The dietary diversity questionnaire represents a rapid, user-friendly and easily administered low-cost assessment tool [13]. In this study, we aimed to investigate the association between dietary diversity and food variety with micronutrient inadequacy among Chinese children, using data from the 2011 China Health and Nutrition Survey (CHNS) [14]. We hypothesized that less dietary diversity and lower food variety were associated with dietary micronutrient inadequacy in children.

## 2. Materials and Methods

### 2.1. Study Population

CHNS data in 2011 was used for analysis. CHNS is a large-scale ongoing longitudinal survey, which was conducted in 218 communities in nine diverse provinces starting from 1989 on the basis of substantial variation in geography, economic development and public resources [14]. In 2011, three megacities were sampled and added to CHNS to improve national representation. Using a multistage random cluster sampling method, more than ten thousand subjects were involved from urban and rural areas. Survey procedures have been described elsewhere [14,15]. All household members were invited into the survey except for those who were unable to recall the answers to the questionnaires or reluctant to participate. For pre-school aged and school aged children, subjects were further enrolled from kindergartens or schools if the projected sample numbers were not achieved from the households approached. The original sample size from the CHNS database was 2086 for children aged 3–17 years. There remained 2043 subjects after omitting those with aberrant energy intake. Finally, after excluding 31 subjects with null key items, such as weight, height, region, gender and age, a total of 2012 subjects were included into the analysis.

The study was conducted in accordance with the Declaration of Helsinki, and the survey protocols were approved by the Institutional Review Committee of the University of North Carolina at Chapel Hill, the National Institute for Nutrition and Health, and the Chinese Center for Disease Control and Prevention. All children and their parents provided their informed consent for inclusion before they participated in the study [14,15]. 

### 2.2. Dietary Survey and Urbanization Level

Trained interviewers from local Centers for Disease Control and Prevention visited the households to collect the information on food consumption using the 24-h dietary recall method over three consecutive days (including two weekdays and one weekend day). For children younger than 12 years, the mother or a caregiver who handled food preparation and feeding in the household was asked to report all foods the children consumed at home and outside the home during the past 24 h. For children over 12 years old, they recalled food consumption independently. Data around edible oils, salt, and condiments consumption was collected by the weighing method at the household level. The dietary assessment was based on the combined data from three consecutive 24-h recalls and household weighing method. Detailed methods of the dietary survey can be seen elsewhere [5].

To develop a validated multidimensional measure of modernization and urbanization, the CHNS team identified 12 additional demographic components to be collected including population density, economic activity, traditional markets, modern markets, transportation infrastructure, sanitation, communications, housing, education, diversity, health infrastructure and social services. These 12 components were collected using questionnaires and measured at the time of survey in each community. Furthermore, a secondary indicator, which was created by the CHNS team based on the data from the 12 components, was used in both multiple linear regression and logistic regression models as an adjustment variable to control for the effect of variation at the community level.

### 2.3. DDS and Food Variety Scores (FVS) Calculation

DDS and FVS were used to assess dietary diversity and food variety. Since DDS and FVS are supposed to reflect the probability of micronutrient adequacy of the diet, the fats and oils, condiments, sugar and wine data, which do not contribute to the essential micronutrient density of the diet, were not included in the DDS and FVS calculation, as per the recommendations of the Food and Agriculture Organization (FAO) guidelines [13].

DDS was defined as the number of food groups consumed over a period of three days of dietary recall. According to the 2016 Chinese Dietary Guideline [6], with consideration of the most common food groups consumed for Chinese children, the following nine food groups were included to construct DDS: (1) cereals, potatoes and starches; (2) fresh vegetables and pure vegetable juice (excluding pickled vegetables); (3) fresh fruits and pure fruit juice (excluding preserved fruits); (4) legumes and nuts; (5) meats (including pork, beef, poultry and organs); (6) fish (including seafood, freshwater fish and aquatic products); (7) eggs; (8) milk; and (9) moderation foods (including sweet or oily cookies, salty snacks, cakes, western fast foods, puffed food, cream and beverages). DDS was calculated by summing the number of unique food groups consumed by the child during the survey days. One point was awarded to each food group if a participant consumed any food in any of the above groups. Consuming different food items from the same group would not be counted repeatedly. An all-inclusive DDS calculation method was used without a minimum intake for the food group. The total score was the sum of the scores for the nine food groups and the maximum score possible was 9. A DDS below the recommendation was defined as a score lower than 8, in line with the Chinese Dietary Guideline (CDG) [6].

As indicated in Fernandez’s study, overall food variety, and food variety in certain food groups, could predict nutritional status in children [16]. We further assessed food variety by using an overall FVS (OFVS), FVS of the fruit and vegetable group (FVS_FV) and FVS of the animal food group (FVS_AF). OFVS was assigned based on the total number of food items of the whole diet consumed during the survey days. One point was given to OFVS for each consumed food item. Scores were assigned to FVS_FV and FVS_AF with the same method. Animal foods were defined as meat, fish, egg and milk. The three FVSs and DDS were further divided into low, medium and high category according to their tertiles.

### 2.4. Assessment of Nutrients Adequacy and Inadequacy

In order to ensure the reliability of the data, aberrant energy intake was identified and omitted before the dietary assessment. Aberrant energy intake was regarded as less than 500 kcal or more than 5000 kcal per day after being converted into “standard person” and was based on the method used in the National Nutrition and Health Surveillance report in 2010–2013 [3]. Average daily micronutrient intake was estimated based on individual food intake data over a three-day period and the Chinese Composition Table (2009 version) [17]. To determine micronutrient adequacy, the nutrient adequacy ratio (NAR) was used for each of the following 14 micronutrients: vitamin A, vitamin C, α-vitamin E, thiamin, riboflavin, calcium, iron, zinc, selenium, niacin, phosphorus, sodium, magnesium and potassium. For each individual, NAR was calculated as the intake of a given nutrient divided by its recommended nutrient intake (RNI). RNI values were based on the latest Chinese Dietary Reference Intakes (DRIs) [18]. Adequate intake (AI) was used alternatively for NAR calculation for those nutrients without RNI, such as vitamin E, sodium and potassium. Similarly, the NAR of energy was calculated as the intake of energy divided by the recommendation (estimated energy requirement, EER) [18] at an individual level. Theoretically, the value of NAR for energy and nutrients could be under, greater than, or equal to 1. In this paper, NAR was truncated at 1 so that a nutrient with a high NAR could not compensate for a nutrient with a low NAR [19]. The mean adequacy ratio (MAR, %), which reflects the adequacy of the overall diet, was defined as the sum of NAR for each micronutrient divided by the number of all involved micronutrients. A value of 1 is ideal for both NAR and MAR since it means that the intake meets the requirement. 

A short-cut method, simply calculating the proportion of intakes that are below the estimated average requirement (EAR), was used to estimate the prevalence of inadequacy of each micronutrient [20,21]. A cut-off point of 0.75 was used for MAR to define overall micronutrient inadequacy (OMI), based on the recommendation in the study by Hatløy et al. [19], which was also used in the study by Schuette et al. [22].

### 2.5. Anthropometric Measures

Standard procedures were followed to conduct anthropometric measurements by well-trained examiners. Weight was measured to the nearest 0.1 kg in light clothing by using the calibrated beam scale. Height was measured to the nearest 0.1 cm without shoes by using a portable stadiometer. Body mass index (BMI) was calculated as weight in kilograms divided by the square of height in meters and expressed as kg/m^2^. HAZ (height for age Z-score) and BAZ (BMI for age Z-score) were computed by WHO Anthro and Anthroplus software. For 3–5 year old children, wasting was defined as WHZ (weight for height Z-score) ≤ −2 and overweight and obesity was defined as BAZ ≥ 1, according to WHO criteria [23,24]. For 6–17 year old children, wasting, overweight and obesity was diagnosed using the age and sex specific BMI cut-off points recommended in latest screening standards released by the National Health Commission of the People’s Republic of China [25,26]. 

### 2.6. Statistical Analysis

Basic data cleaning had been done by the CHNS team before the datasets were uploaded online, including corrections to identification variables and the household list deletion of duplicate or blank records, recoding out-of-range values to missing status (only values that were clearly impossible were recorded to missing). Continuous variables such as diversity or variety scores, HAZ, BAZ, NAR of micronutrient intake were presented as mean and standard deviation (SD). Categorical variables were presented as case and percentage. The Student *t*-test was used to test the differences of the means of continuous variables and Chi-square was used to test the distribution of the percentage of categorical variables between two age groups. For the NAR of each micronutrient and the MAR among low, medium and high dietary diversity or variety categories, the general linear model was used to estimate the overall statistical difference and the Student–Newman–Keuls method was applied to test the differences between two categories.

Multiple linear regression was conducted to assess associations of nutrient adequacy with DDS and FVSs, in which the NAR of each micronutrient and the MAR were regarded as the lone dependent variable, while DDS and FVSs (as continuous variables) were regarded as independent variables in each model. The logistic regression model was employed to explore the association of nutrient inadequacy with DDS and FVS categories. Inadequacy of each nutrient and OMI were worked as dependent variables while three categories of each dietary variety/food variety score were worked as independent variables. We further adjusted age, gender, urbanization level, daily energy intake and BAZ in both multiple linear regression models and logistic regression models. *p* < 0.05 was considered statistically significant, and SAS package version 9.3 (SAS Institute Inc., Cary, NC, USA) was used for statistical analyses.

## 3. Results

### 3.1. General Information

Table 1 shows the characteristics of the study population. A total of 1485 children aged 3–12 years old and 527 adolescents aged 13–17 years were included for analysis. The mean age was 9.2 years with a SD of 4.1. For the total population, the mean and SD of OFVS, DDS and MAR were 16.7 ± 6.4, 6.1 ± 1.7 and 0.8 ± 0.2, respectively. Mean HAZ (*p* < 0.01), BAZ (*p* < 0.01) and MAR (*p* < 0.05) scores were significantly higher in children than in adolescents. By contrast, the mean values of OFVS (*p* < 0.05), FVS_FV (*p* < 0.05) and FVS_AF (*p* < 0.01) were significantly lower in children compared to adolescents. Compared with children, adolescents had higher total energy and all micronutrient intake (*p* < 0.01). however, their energy and micronutrient (vitamin A, thiamin, riboflavin, α-vitamin E, selenium, magnesium and potassium) adequacy rates were lower than those for children, as demonstrated by comparing the average NAR level for each group (*p* < 0.01). 

**Figure 1:**
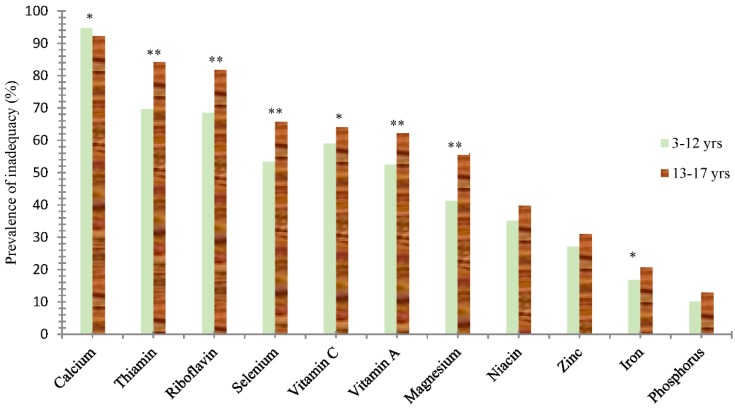
Prevalence of inadequacy of micronutrients. * Significant difference between two age groups, *p* < 0.05, ** *p* < 0.01.

The prevalence of inadequate intake of most micronutrients except niacin, zinc and phosphorus was significantly higher in adolescents than those in children (Figure 1).

With regard to the differences of DDS and the three FVSs between higher and lower MAR groups in urban and rural areas, results are shown in Table A1. 

### 3.2. Association of DDS and FVS with Nutrient Inadequacy

For energy and each micronutrient, the mean NAR was significantly higher in high DDS or high FVS groups (*p* < 0.05), and a similar trend was also found for MAR means (Table 2). 

As shown in Table 3, the DDS was positively associated with MAR, as well as with the NAR of all micronutrients (*p* < 0.05) with the exception of sodium. Similar positive associations were also seen between OFVS, FVS_FV, FVS_AF with MAR and NAR of all micronutrients except sodium (Table 3). 

Compared with the low DDS group, subjects with a high DDS had a lower risk of micronutrient inadequacy, with odd ratios (ORs) ranging between 0.1and 0.7. Similarly, compared with low OFVS, subjects with high OFVS were at lower risk of micronutrient inadequacy of all micronutrients except for calcium, with the OR ranging between 0.1 and 0.7. Similar associations were also found for FVS_FV (except calcium) and FVS_AF (except magnesium) (Table 4). 

## 4. Discussion

This present study assessed the association of dietary diversity and food variety with micronutrient inadequacy among Chinese children aged 3–17 years using national representative data. Results showed that the adequacy rates were low for some micronutrients in Chinese children, such as calcium, vitamin A, thiamin, riboflavin, vitamin C, vitamin E, and selenium. In addition, we have demonstrated that both dietary diversity and food variety are positively associated with dietary micronutrient intake, and negatively associated with micronutrient inadequacy. These findings indicate that dietary diversity and food variety could be used for screening purposes to identify the risk of micronutrient inadequacy in Chinese children.

The value of DDS in our study was determined to be 6.11, and it is higher than those reported in two other similar studies carried out with Chinese children which reported DDS values of 5.60 and 4.18 [7,27]. Differences in the methodology used to calculate DDS are likely reasons for the lack of alignment between the DDS values reported for each study. For instance, in the 2010 study by Li et al., which reported a DDS of 4.18 for children aged 2–7 years, researchers calculated the DDS using three 24-h recalls based on 13 food categories (compared to nine in the present study) with points allocated for at least 25 g of consumption for each food sub-category [27]. A more recent study in 2017, which reported a DDS of 5.60 for children aged 6–12 years, determined the DDS based on 10 food groups using one 24-h dietary recall data set with points being allocated only when a threshold intake of 10g or more for each food sub-category was met [7]. DDS calculations that have a threshold of food intake will ignore sub-categories for which dietary intake is under the threshold and therefore will be likely to have lower DDS values. In our study, an all-inclusive DDS calculation method was used without a minimum intake for each food sub-category and therefore, would be expected to return relatively higher DDS values when compared to threshold-based calculation methods. Currently there is no unified cutoff point for setting thresholds for DDS calculation, making direct comparisons between studies problematic. Further differences in methodology include the timeframe across which dietary information is collected. Dietary diversity over a three day period (as in this present study) will undoubtedly be higher than that observed during one day, which might be another reason for the higher DDS in our study. Worldwide there is also a large variety of DDS and FVS values reported among studies, which again appears to be due to differences in how the parameters used have been defined and calculated [8,9,19]. These differences include variations in definitions of the food groups (e.g., the number and make up of food groups used, or the use of food codes or even food ingredients in some studies), applying thresholds of intake for each food group, and different methods for extracting diversity and variety data, such as using one 24-h recall period versus three or seven 24-h recall periods, or even food frequency questionnaires (FFQ). 

In the current study, both DDS and OFVS were positively associated with the MAR and NAR for most micronutrients, which was consistent with other studies [19,28,29,30]. Steyn et al., who assessed the association between dietary diversity and nutrient adequacy in 1–8 year old children in South Africa, demonstrated a high correlation between MAR and both FVS (r = 0.726, *p* < 0.0001) and DDS (r = 0.657; *p* < 0.0001). DDS was associated with the NAR of 11 micronutrients (vitamin A, vitamin B6, vitamin B12, thiamin, riboflavin, folate, niacin, vitamin C, calcium, iron and zinc) with Pearson correlation coefficients ranging from 0.1 to 0.6 [28]. Similarly, dietary diversity was found to be a good predictor of either micronutrient density of the diet in 6- to 23-month old children in Madagascar [29], or nutritional adequacy in 10–18 year old adolescents in Iran [31]. Several reasons may explain the positive associations. First, higher dietary diversity reflects that an individual has more access to a variety of foods and therefore is likely to have higher food consumption and nutrient intake. In fact, further analysis of the data in the present study verified that the food consumption increased significantly with increasing DDS. Second, higher DDS has been related to more consumption of healthy and higher nutrient density foods, such as fruit, vegetables and whole grains [32], which could partly explain the higher micronutrient intake.

In the present study, FVS_FV and FVS_AF were positively correlated with overall micronutrient adequacy and NAR for most micronutrients, and additionally, subjects in the high FVS_FV or FVS_AF groups had a lower risk of inadequate intake for most micronutrients. This observation makes sense in light of the nutritional qualities of fruits and vegetables, which are rich in vitamins and minerals, and are recommended in the dietary guidelines of many countries [6]. Additionally, different fruits and vegetables are often complementary and irreplaceable because the nutrient profile varies greatly from item to item [6]. For example, red and yellow fruits are rich in carotene while bananas are rich in potassium. Green leafy vegetables are rich in vitamin B_1_, vitamin B_2_ and vitamin C, while carrots are rich in carotene. The vitamin B family and vitamin D are generally higher in fungi and algae than in other vegetables [17]. Again, the positive correlation of FVS_AF is also easily explained as animal foods are an excellent source of high quality protein and several minerals and vitamins like magnesium, iron, zinc, vitamin E and B- vitamins. Because of these properties, nutrient-rich foods such as animal foods or foods fortified with micronutrients are recommended by the World Health Organization (WHO) to improve micronutrient intake [33]. Taken together, higher FVS_VS and higher FVS_AF mean there is a good chance of obtaining micronutrient variety and balance.

Regarding the best methodologies for dietary and nutrition assessment for individuals, generally the daily food and nutrients intake and dietary quality assessment methods are ideal for drawing a clear structure of nutritional status. However, these methods become more complex and time-consuming as study population sizes are increased, making them less relevant for use in those circumstances. As an alternative, individual dietary diversity scores have promise as a rapid and efficient means to estimate nutrient adequacy of an individual’s diet, as has been shown in this present study. For example, health workers can do a quick 24-h recall to ascertain a child’s DDS. Caregivers can be simply asked to answer the total number of food items of animal foods, or fruit and vegetable foods. If the children have a DDS value less than five, or FVS_FV value less than five, or FVS_AF value less than three, they will most probably be at risk of inadequate micronutrient intake. Due to limited public health resources, health workers require simple, quick and reasonably accurate indicators to find the most vulnerable subjects at high risk of nutrient inadequacy. The use of DDS and FVS indicators make it possible to screen and discriminate high-risk subjects easily during large scale surveillance and may be of particular relevance for use with children. A more detailed nutritional assessment can then be further conducted for the limited number of vulnerable children identified using these methods if needed. 

The present study had several strengths. First, two sets of diversity measures, dietary diversity (based on food groups) and food variety (based on individual foods), were concurrently included in this study. Second, daily energy intake and urbanization level were adjusted in multivariate regressions to reduce the risk of bias. There are also limitations that should be noted. Firstly, this was a cross-sectional study, so causal relationships cannot be determined. Secondly, the dietary diversity and food variety scores as determined in this study are likely to result in an underestimate of the children with undernutrition. This is because they were calculated based on three 24-h recalls with no limit on required intake thresholds, likely resulting in inflated scores for DDS and FVSs when compared to calculations using a 24-h recall methodology. 

## 5. Conclusions

This study revealed that dietary diversity, FVS_FV and FVS_AF were positively associated with dietary micronutrient intake, and negatively associated with micronutrient inadequacy. Dietary diversity and food variety measurements could be used in large scale screening for identifying the risk of micronutrient inadequacy in Chinese children.

## Figures and Tables

**Table 1:** Basic characteristics of the study population (%).

	Children (3–12 years, *n* = 1485)		Adolescents (13–17 years, *n* = 527)		Total (*n* = 2012)	
Region						
Urban (*n*, %)	499, 33.60		225, 42.69		724, 35.98	
Rural (*n*, %)	986, 66.40		302, 57.31		1288, 64.02	
*p* for Chi-square	0.0002					
Gender						
Boys (*n*, %)	775, 52.19		269, 51.04		1044, 51.89	
Girls (*n*, %)	710, 47.81		258, 48.96		968, 48.11	
*p* for Chi-square	0.65					
Anthropometric measures						
HAZ (mean, SD)	0.37, 1.40		−0.11 **, 1.09		0.25, 1.35	
BAZ (mean, SD)	0.13, 1.45		−0.06 **, 1.13		0.08, 1.37	
Wasting (*n*, %)	147, 9.90		49, 9.30		196, 9.74	
Overweight and Obesity (*n*, %)	323, 21.75		80, 15.18 **		403, 20.03	
Dietary diversity and food variety						
DDS below recommendation (*n*, %)	1151, 77.51		406. 77.04		1557, 77.39	
FVS below recommendation (*n*, %)	353, 23.77		105, 19.92		458, 22.76	
FVS_FV below recommendation (*n*, %)	223, 15.02		71, 13.47		294, 14.61	
DDS (mean, SD)	6.10, 1.67		6.14, 1.67		6.11, 1.67	
OFVS (mean, SD)	16.53, 6.35		17.28 *, 6.32		16.73, 6.35	
FVS_FV (mean, SD)	6.51, 3.12		6.84 *, 3.07		6.60, 3.11	
FVS_AF (mean, SD)	3.85, 2.41		4.21 **, 2.65		3.95, 2.48	
	**NAR**	**Intake**	**NAR**	**Intake**	**NAR**	**Intake**
NAR of nutrient and nutrient intake ^†^						
Energy	0.83, 0.18	1533.21, 558.42	0.77 **, 0.20	2049.58 **, 755.70	0.81, 0.19	1668.50, 656.54
Vitamin A	0.66, 0.32	434.36, 495.44	0.59 **, 0.31	600.71 **, 1365.02	0.64, 0.32	477.93, 820.87
Thiamine	0.65, 0.24	0.64, 0.34	0.60 **, 0.22	0.85 **, 0.37	0.64, 0.23	0.70, 0.36
Riboflavin	0.66, 0.26	0.65, 0.38	0.59 **, 0.23	0.82 **, 0.45	0.64, 0.25	0.69, 0.40
Vitamin C	0.67, 0.30	54.69, 45.58	0.65, 0.29	79.23 **, 94.82	0.67, 0.30	61.11, 63.25
Calcium	0.36, 0.22	333.06, 231.11	0.40 **, 0.22	430.06 **, 258.23	0.37, 0.22	358.47, 242.23
Iron	0.90, 0.16	14.81, 8.89	0.89, 0.16	20.05 **, 11.18	0.90, 0.16	16.18, 9.82
Zinc	0.89, 0.17	7.55, 3.42	0.87, 0.18	10.19 **, 4.22	0.89, 0.17	8.24, 3.83
Selenium	0.75, 0.25	33.76, 20.68	0.69 **, 0.25	45.85 **, 25.60	0.74, 0.25	36.92, 22.70
α-vitamin E	0.73, 0.29	8.26, 6.58	0.64 **, 0.31	11.13 **, 9.87	0.71, 0.30	9.01, 7.68
Niacin	0.84, 0.21	10.33, 5.43	0.84, 0.21	14.68 **, 8.03	0.84, 0.21	11.47, 6.50
Phosphorus	0.97, 0.10	697.98, 312.14	0.95 *, 0.12	935.39 **, 367.00	0.96, 0.11	760.17, 343.56
Sodium	0.97, 0.14	4.37, 3.36	0.97, 0.15	6.10 **,8.02	0.97, 0.14	4.82, 5.07
Magnesium	0.84, 0.19	203.20, 91.22	0.78 **, 0.20	278.78 **, 127.80	0.83, 0.19	223.00, 107.32
Potassium	0.80, 0.21	1.35, 0.70	0.75 **, 0.21	1.82 **, 1.01	0.79, 0.21	1.47, 0.82
MAR (mean, SD)	0.76, 0.15		0.73 *, 0.15		0.75, 0.15	

HAZ, height for age Z-score; BAZ, BMI for age Z-score; DDS, dietary diversity score; FVS, food variety score; OFVS, overall fruit and vegetables score, FVS_FV, food variety scores of fruits and vegetables; FVS_AF, food variety scores of animal foods; NAR, nutrient adequacy ratio; MAR, mean adequacy ratio; SD, standard deviation. ^†^ NAR of micronutrient and micronutrient intake were expressed as mean and standard deviation. Unit of nutrients: kcal for energy, µg retinol activity equivalent for vitamin A, µg for selenium, g for potassium, mg for all the other micronutrients. * Significant difference between two age groups, *p* < 0.05, ** *p* < 0.01.

**Table 2:** NAR of nutrients and MAR among children in low, medium and high category of DDS and three FVSs ^‡^ (*n* = 2012).

	DDS						OFVS						FVS_FV						FVS_AF					
	Low (<5)		Medium (5–6)		High (≥7)		Low (<13)		Medium (13–19)		High (≥19)		Low (<5)		Medium (5–7)		High (≥8)		Low (<3)		Medium (3–4)		High (≥5)	
	Mean	SD	Mean	SD	Mean	SD	Mean	SD	Mean	SD	Mean	SD	Mean	SD	Mean	SD	Mean	SD	Mean	SD	Mean	SD	Mean	SD
NAR																								
Energy	0.7 ^a^	0.2	0.8 ^b^	0.2	0.9 ^c^	0.2	0.7 ^a^	0.2	0.8 ^b^	0.2	0.9 ^c^	0.2	0.8 ^a^	0.2	0.8 ^b^	0.2	0.9 ^c^	0.2	0.7 ^a^	0.2	0.7 ^b^	0.2	0.8 ^c^	0.2
Vitamin A	0.4 ^a^	0.3	0.6 ^b^	0.3	0.7 ^c^	0.3	0.5 ^a^	0.4	0.6 ^b^	0.3	0.8 ^c^	0.3	0.5 ^a^	0.3	0.6 ^b^	0.3	0.8 ^c^	0.3	0.3 ^a^	0.3	0.5 ^b^	0.3	0.7 ^c^	0.3
Thiamine	0.5 ^a^	0.2	0.6 ^b^	0.2	0.7 ^c^	0.2	0.6 ^a^	0.2	0.6 ^b^	0.2	0.7 ^c^	0.2	0.6 ^a^	0.2	0.6 ^b^	0.2	0.7 ^c^	0.2	0.5 ^a^	0.2	0.5 ^b^	0.2	0.7 ^c^	0.2
Riboflavin	0.4 ^a^	0.2	0.6 ^b^	0.2	0.8 ^c^	0.2	0.5 ^a^	0.2	0.6 ^b^	0.2	0.8 ^c^	0.2	0.5 ^a^	0.2	0.6 ^b^	0.2	0.8 ^c^	0.2	0.3 ^a^	0.2	0.4 ^b^	0.2	0.7 ^c^	0.2
Vitamin C	0.5 ^a^	0.3	0.7 ^b^	0.3	0.8 ^c^	0.3	0.6 ^a^	0.3	0.7 ^b^	0.3	0.8 ^c^	0.3	0.5 ^a^	0.3	0.7 ^b^	0.3	0.8 ^c^	0.2	0.5 ^a^	0.3	0.6 ^b^	0.3	0.7 ^c^	0.3
Calcium	0.2 ^a^	0.2	0.3 ^b^	0.2	0.5 ^c^	0.2	0.3 ^a^	0.2	0.3 ^b^	0.2	0.5 ^c^	0.2	0.3 ^a^	0.2	0.3 ^b^	0.2	0.5 ^c^	0.2	0.2 ^a^	0.2	0.3 ^b^	0.2	0.4 ^c^	0.2
Iron	0.8 ^a^	0.2	0.9 ^b^	0.2	1.0 ^c^	0.1	0.8 ^a^	0.2	0.9 ^b^	0.2	1.0 ^c^	0.1	0.8 ^a^	0.2	0.9 ^b^	0.2	1.0 ^c^	0.1	0.7 ^a^	0.2	0.8 ^b^	0.2	0.9 ^c^	0.1
Zinc	0.8 ^a^	0.2	0.9 ^b^	0.2	0.9 ^c^	0.1	0.8 ^a^	0.2	0.9 ^b^	0.2	1.0 ^c^	0.1	0.8 ^a^	0.2	0.9 ^b^	0.2	0.9 ^c^	0.1	0.7 ^a^	0.2	0.8 ^b^	0.2	0.9 ^c^	0.1
Selenium	0.5 ^a^	0.3	0.7 ^b^	0.2	0.9 ^c^	0.2	0.6 ^a^	0.3	0.8 ^b^	0.2	0.9 ^c^	0.2	0.6 ^a^	0.3	0.7 ^b^	0.3	0.8 ^c^	0.2	0.5 ^a^	0.3	0.6 ^b^	0.3	0.8 ^c^	0.2
α-vitamin E	0.6 ^a^	0.3	0.7 ^b^	0.3	0.8 ^c^	0.3	0.6 ^a^	0.3	0.7 ^b^	0.3	0.8 ^c^	0.3	0.6 ^a^	0.3	0.7 ^b^	0.3	0.8 ^c^	0.3	0.5 ^a^	0.4	0.5 ^b^	0.3	0.7 ^c^	0.3
Niacin	0.7 ^a^	0.3	0.8 ^b^	0.2	0.9 ^c^	0.2	0.8 ^a^	0.2	0.8 ^b^	0.2	0.9 ^c^	0.2	0.8	0.2 ^a^	0.8 ^b^	0.2	0.9 ^c^	0.2	0.6 ^a^	0.2	0.7 ^b^	0.3	0.9 ^c^	0.2
Phosphorus	0.9 ^a^	0.2	1.0 b	0.1	1.0 ^c^	0.0	0.9 ^a^	0.2	1.0 ^b^	0.1	1.0 ^c^	0.0	0.9 ^a^	0.2	1.0 ^b^	0.1	1.0 ^c^	0.1	0.8 ^a^	0.2	0.9 ^b^	0.2	1.0 ^c^	0.1
Sodium	0.9 ^a^	0.2	1.0 ^a^	0.1	1.0 ^b^	0.1	1.0 ^a^	0.2	1.0 ^a^	0.1	1.0 ^b^	0.1	1.0 ^a^	0.2	1.0 ^a^	0.1	1.0 ^b^	0.1	0.9 ^a^	0.3	1.0 ^a^	0.2	1.0 ^b^	0.1
Magnesium	0.7 ^a^	0.2	0.8 ^b^	0.2	0.9 ^c^	0.2	0.8 ^a^	0.2	0.8 ^b^	0.2	0.9 ^c^	0.2	0.8 ^a^	0.2	0.8 ^b^	0.2	0.9 ^c^	0.2	0.7 ^a^	0.2	0.8 ^b^	0.2	0.8 ^c^	0.2
Potassium	0.6 ^a^	0.2	0.8 ^b^	0.2	0.9 ^c^	0.2	0.7 ^a^	0.2	0.8 ^b^	0.2	0.9 ^c^	0.2	0.7 ^a^	0.2	0.8 ^b^	0.2	0.9 ^c^	0.2	0.6 ^a^	0.2	0.7 ^b^	0.2	0.8 ^c^	0.2
MAR ^‖^	0.6 ^a^	0.2	0.7 ^b^	0.2	0.8 ^c^	0.1	0.6 ^a^	0.2	0.7 ^b^	0.2	0.8 ^c^	0.1	0.6 ^a^	0.2	0.7 ^b^	0.2	0.8 ^c^	0.1	0.6 ^a^	0.2	0.6 ^b^	0.2	0.8 ^c^	0.1

DDS, dietary diversity score; FVS, food variety score; OFVS, overall fruit and vegetables score, FVS_FV, food variety scores of fruits and vegetables; FVS_AF, food variety scores of animal foods; NAR, nutrient adequacy ratio; MAR, mean adequacy ratio. ^‡^ Results were described as mean with standard deviation. ^‖^ The MAR value for low, medium and high FVS_AF categories is 0.55, 0.64 and 0.78, respectively. ^a^, ^b^, ^c^ Mean values with unlike superscript letters were significantly different among low, medium and high diversity/variety group (*p* < 0·05).

**Table 3:** Association of DDS and FVS with NARs and MAR using linear regression model ^§^ (*n* = 2012).

	DDS			OFVS			FVS_FV			FVS_AF		
	Β	SE	*p*	Β	SE	*p*	Β	SE	*p*	Β	SE	*p*
NAR												
Vitamin A	0.054	0.005	<0.0001	0.013	0.001	<0.0001	0.032	0.002	<0.0001	0.040	0.003	<0.0001
Thiamine	0.011	0.003	0.000	0.003	0.001	<0.0001	0.010	0.002	<0.0001	0.010	0.002	<0.0001
Riboflavin	0.062	0.003	<0.0001	0.014	0.001	<0.0001	0.022	0.001	<0.0001	0.043	0.002	<0.0001
Vitamin C	0.040	0.004	<0.0001	0.014	0.001	<0.0001	0.040	0.002	<0.0001	0.017	0.003	<0.0001
Niacin	0.031	0.003	<0.0001	0.006	0.000	<0.0001	0.011	0.001	<0.0001	0.030	0.002	<0.0001
α-vitamin E	0.013	0.004	0.001	0.003	0.001	0.002	0.007	0.002	0.001	0.009	0.003	0.002
Calcium	0.043	0.003	<0.0001	0.009	0.001	<0.0001	0.010	0.001	<0.0001	0.026	0.002	<0.0001
Iron	0.017	0.002	<0.0001	0.004	0.000	<0.0001	0.008	0.000	<0.0001	0.009	0.001	<0.0001
Zinc	0.023	0.002	<0.0001	0.004	0.001	<0.0001	0.008	0.001	<0.0001	0.018	0.001	<0.0001
Selenium	0.048	0.001	<0.0001	0.011	0.001	<0.0001	0.009	0.002	<0.0001	0.032	0.002	<0.0001
Phosphorus	0.016	0.001	<0.0001	0.003	0.000	<0.0001	0.004	0.000	<0.0001	0.007	0.001	<0.0001
Sodium	0.000	0.002	0.754	−0.001	0.001	0.061	−0.003	0.001	0.018	0.001	0.002	0.375
Magnesium	0.015	0.002	<0.0001	0.004	0.000	<0.0001	0.009	0.001	<0.0001	0.004	0.002	0.024
Potassium	0.036	0.003	<0.0001	0.009	0.001	<0.0001	0.017	0.001	<0.0001	0.018	0.002	<0.0001
MAR	0.029	0.002	<0.0001	0.007	0.000	<0.0001	0.013	0.001	<0.0001	0.019	0.001	<0.0001

DDS, dietary diversity score; FVS, food variety score; OFVS, overall fruit and vegetables score, FVS_FV, food variety scores of fruits and vegetables; FVS_AF, food variety scores of animal foods; NAR, nutrient adequacy ratio; MAR, mean adequacy ratio; B, value of parameter estimate; SE, standard error. ^§^ NAR of each nutrient and MAR were regarded as the lone dependent variable while DDS and three FVSs were as independent variables in each model. Confounders included age, gender, urbanization level, daily energy intake and BAZ were adjusted in each model.

**Table 4:** Association of dietary diversity and food variety with micronutrient inadequacy ^††^ (*n* = 2012).

	DDS (Ref.: low, <5)				OFVS (Ref.: low, <13)				FVS_FV (Ref.: low, <5)				FVS_AF (Ref.: low, <3)			
	Medium (5–6)		High (≥7)		Medium (13–19)		High (≥19)		Medium (5–7)		High (≥8)		Medium (3–4)		High (≥5)	
	OR	95% CI	OR	95% CI	OR	95% CI	OR	95% CI	OR	95% CI	OR	95% CI	OR	95% CI	OR	95% CI
Inadequacy of micronutrient																
Vitamin A	0.7	0.5, 0.9	0.4	0.3, 0.6	0.9	0.7, 1.1	0.5	0.4, 0.6	0.6	0.5, 0.7	0.3	0.2, 0.4	0.5	0.4, 0.7	0.3	0.2, 0.4
Thiamine	0.7	0.5, 1.1	0.7	0.5, 1.1	0.8	0.6, 1.1	0.7	0.5, 0.9	0.8	0.6, 1.1	0.5	0.3, 0.7	0.8	0.5, 1.1	0.4	0.4, 0.7
Riboflavin	0.3	0.2, 0.5	0.1	0.07, 0.2	0.4	0.3, 0.5	0.1	0.1, 0.2	0.6	0.4, 0.8	0.2	0.18, 0.3	0.2	0.1, 0.4	0.1	0.1, 0.2
Vitamin C	0.7	0.5, 0.9	0.5	0.3, 0.6	0.8	0.6, 1.0	0.5	0.4, 0.6	0.5	0.4, 0.7	0.3	0.2, 0.3	0.8	0.6, 1.0	0.7	0.5, 0.9
Calcium	1.5	0.6, 3.8	0.6	0.2, 1.4	0.9	0.4, 2.0	0.5	0.2, 1.0	1.1	0.5, 2.5	0.7	0.4, 1.2	1.1	0.5, 2.4	0.3	0.2, 0.7
Iron	0.6	0.5, 0.9	0.3	0.2, 0.5	0.7	0.5, 0.9	0.4	0.3, 0.5	0.9	0.7, 1.3	0.4	0.3, 0.7	0.8	0.6, 1.2	0.5	0.3, 0.7
Zinc	0.6	0.5, 0.9	0.3	0.2, 0.5	1.0	0.7, 1.3	0.5	0.3, 0.7	0.9	0.7, 1.2	0.4	0.3, 0.6	0.5	0.4, 0.6	0.1	0.1, 0.2
Selenium	0.7	0.5, 0.9	0.2	0.1, 0.3	0.4	0.3, 0.5	0.3	0.2, 0.4	0.7	0.5, 0.9	0.5	0.4, 0.7	0.4	0.3, 0.6	0.2	0.1, 0.2
Niacin	0.6	0.4, 0.8	0.3	0.3, 0.5	0.8	0.6, 1.1	0.4	0.3, 0.6	0.7	0.6, 1.0	0.5	0.4, 0.7	0.4	0.3, 0.5	0.2	0.1, 0.2
Phosphorus	0.4	0.3, 0.6	0.1	0.1, 0.3	0.8	0.5, 1.1	0.2	0.1, 0.4	0.6	0.4, 0.9	0.2	0.1, 0.3	0.7	0.5, 1.1	0.2	0.1,0.3
Magnesium	0.9	0.7, 1.3	0.6	0.4, 0.8	1.1	0.8, 1.5	0.6	0.5, 0.9	0.8	0.6, 1.0	0.5	0.4, 0.7	1.0	0.8,1.4	0.8	0.6, 1.2
OMI	0.4	0.3, 0.6	0.2	0.1, 0.3	0.5	0.4, 0.7	0.2	0.1, 0.3	0.5	0.4, 0.7	0.2	0.1, 0.3	0.4	0.3, 0.5	0.2	0.1, 0.2

DDS, dietary diversity score; FVS, food variety score; OFVS, overall fruit and vegetables score, FVS_FV, food variety scores of fruits and vegetables; FVS_AF, food variety scores of animal foods. OMI, overall micronutrient inadequacy. ^††^ inadequacy of each micronutrient and OMI were worked as dependent variables while dietary variety/food variety scores were worked as independent variables. Confounders included age, gender, urbanization level, daily energy intake and BAZ were adjusted in each model.

## Appendix A

**Table A1:** Dietary diversity score, food variety score by region and MAR level¶.

	Urban (*n* = 724)				Rural (*n* = 1288)				All (*n* = 2012)			
	MAR ≥ 0.75		MAR < 0.75		MAR ≥ 0.75		MAR < 0.75		MAR ≥ 0.75		MAR < 0.75	
	Mean	SD	Mean	SD	Mean	SD	Mean	SD	Mean	SD	Mean	SD
DDS	7.3	1.3	5.8	1.6	6.3	1.4	5.1	1.6	6.7	1.5	5.3	1.6
OFVS	21.5	5.9	16.1	5.6	17.0	5.9	13.1	4.8	18.9	6.3	14.0	5.2
FVS_FV	8.2	3.0	5.9	2.8	7.1	3.1	5.2	2.5	7.6	3.1	5.4	2.6
FVS_AF	6.0	2.4	3.8	2.1	3.9	2.2	2.5	1.8	4.8	2.5	2.9	2.0

MAR, mean adequacy ratio; DDS, dietary diversity score, OFVS, overall fruit and vegetables score, FVS_FV, food variety scores of fruits and vegetables; FVS_AF, food variety scores of animal foods. ¶ Student T-test was used for the difference of the means between MAR ≥ 0.75 and MAR < 0.75group. There were significant differences for DDS and four FVS between two MAR groups in urban, rural as well as in nation level, *p* < 0.01.
